# Unilateral Ptosis and Bulbar Symptoms as the Initial Presentation of Late-Onset Acetylcholine Receptor Antibody-Positive Myasthenia Gravis Mimicking Acute Ischemic Stroke in an 82-Year-Old Man

**DOI:** 10.7759/cureus.99832

**Published:** 2025-12-22

**Authors:** Sughra M Mangrio, Sadia Faisal, Zoya Malik, Ahmed Naeem, Urooj Saeed

**Affiliations:** 1 General Internal Medicine, Russells Hall Hospital, Dudley, GBR; 2 Stroke Medicine, Russells Hall Hospital, Dudley , GBR; 3 Stroke Medicine, Russells Hall Hospital, Dudley, GBR

**Keywords:** acetylcholine receptor antibody, bulbar symptoms, elderly patient, late-onset myasthenia, myasthenia gravis, neuromuscular junction disorder, ocular myasthenia, speech disorders, stroke mimic, unilateral ptosis

## Abstract

Myasthenia gravis is an autoimmune disorder of the neuromuscular junction. In older adults, it may present with bulbar and ocular symptoms that resemble acute stroke. Ocular myasthenia is typically asymmetric and often bilateral; persistent unilateral ptosis in this context can be misleading. An 82-year-old man with type 2 diabetes mellitus, chronic kidney disease, cataracts, and macular degeneration presented with a two-week history of fatigable dysphagia, slurred speech, and new-onset unilateral left ptosis. Symptoms were worse towards the end of the day. He denied diplopia and limb weakness. On examination, he was alert with marked left ptosis, mild left facial weakness, and normal limb strength and gait. A working diagnosis of ischemic stroke was made. Computed tomography of the head, magnetic resonance imaging of the brain with magnetic resonance angiography, and carotid Doppler ultrasound showed no acute vascular pathology. Speech and language therapy assessment demonstrated moderately dysarthric but functional speech and a safe swallow using compensatory strategies. ENT review was arranged as a safety assessment to exclude structural lesions or throat pathology in view of his recent swallowing difficulty, sensation of food sticking on the left side, and intermittent nasal speech later in the day; flexible nasal endoscopy was normal. Neurology review noted fluctuating bulbar symptoms with isolated unilateral ptosis, normal extraocular movements, and a normal limb examination, raising suspicion for myasthenia gravis. Pyridostigmine was started with rapid improvement in ptosis, speech, and swallowing. By the following day, both eyes were open, and he was seen reading, although transient recurrence of ptosis was observed during reassessment, consistent with fatiguability. Computed tomography of the chest excluded thymoma. During brief withdrawal of pyridostigmine for neurophysiology, unilateral ptosis and dysphagia recurred. Nerve conduction studies and limited single-fibre electromyography were non-diagnostic. Subsequent serology confirmed positive acetylcholine receptor antibodies (13.8) and negative anti-MuSK antibodies. He was treated with pyridostigmine and oral prednisolone with gastric and bone protection and close monitoring of glycaemic control. At follow-up, he remained asymptomatic, with full resolution of unilateral ptosis and bulbar symptoms. Persistent unilateral ptosis with fluctuating bulbar symptoms in an elderly patient, normal neuroimaging, and preserved limb strength should prompt consideration of myasthenia gravis despite an initial stroke pathway. As most cases classically present before the age of 50-60 years, this presentation in an 82-year-old man also illustrates very late-onset acetylcholine receptor antibody-positive disease. This case highlights that unilateral ocular involvement does not exclude myasthenia gravis and that close attention to fatigability and symptom evolution can prevent misdiagnosis and support timely, appropriate treatment.

## Introduction

Myasthenia gravis (MG) is an autoimmune disorder of the neuromuscular junction characterised by fluctuating weakness of skeletal muscles, most often affecting the ocular, bulbar and proximal limb muscles [1]. Classically, many cases present before the age of 50-60 years, but an increasing number of patients are now recognised with late-onset disease in older age groups [1,2].

Ocular involvement is common at presentation and typically includes ptosis and diplopia [1,3]. Ptosis is often asymmetric and may initially be unilateral, which can lead to consideration of alternative diagnoses such as third nerve palsy, Horner syndrome or structural lesions [3,4]. When unilateral ptosis occurs in an elderly patient with vascular risk factors and is accompanied by speech or swallowing difficulty, stroke is frequently suspected.

MG is a recognised stroke mimic, especially when bulbar symptoms such as dysarthria and dysphagia predominate [5-7]. Although ocular involvement in MG is typically asymmetric and often bilateral, early presentations may appear unilateral, which can misleadingly suggest a stroke.

In this setting, normal neuroimaging and preserved limb strength are important clues that should prompt reconsideration of the diagnosis, but recognition may be delayed when symptoms are subtle, fluctuating or when electrophysiological studies are inconclusive.

We report an 82-year-old man with acetylcholine receptor (AChR) antibody-positive MG whose first manifestations were unilateral ptosis and bulbar symptoms, initially managed along a stroke pathway. This case underlines the importance of considering late-onset MG in elderly patients with subacute, fluctuating symptoms and highlights that persistent unilateral ocular involvement does not exclude this diagnosis.

## Case presentation

An 82-year-old man presented with a two week history of progressive swallowing difficulty, slurred speech, and inability to open his left eye. Symptoms worsened with use and as the day progressed. He denied diplopia, limb weakness, dyspnoea, fevers, or other systemic symptoms.

His past medical history included type 2 diabetes mellitus (well controlled prior to admission), chronic kidney disease, vertigo, cataracts, and macular degeneration. He did not smoke and drank alcohol socially.

**Figure 1:**
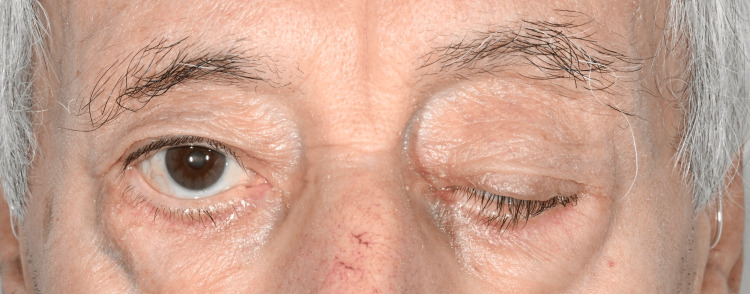
Left upper eyelid ptosis. Clinical photograph at presentation showing marked left upper eyelid ptosis on the left side with preserved eye opening on the right. The image was taken before initiation of pyridostigmine. Published with the patient's written informed consent.

On arrival, he was alert, orientated, and able to provide a clear history. Blood pressure was 103/61 mmHg, and his NEWS score was 2. Speech was intermittently slurred by history but normal at initial assessment. Examination showed marked left upper eyelid ptosis (Figure 1) and mild left facial weakness. Pupils were equal and reactive, extraocular movements were full, and there was no diplopia. Limb tone and power were normal (5/5 throughout) with intact coordination and sensation, and gait was normal. In view of the subacute bulbar symptoms and unilateral facial weakness, a working diagnosis of possible ischemic stroke (partial anterior circulation infarct) was made.

Electrocardiogram showed sinus rhythm. Routine blood tests, including renal profile, were stable. Computed tomography of the head revealed no acute intracranial pathology. Magnetic resonance imaging of the brain with magnetic resonance angiography showed no diffusion restriction or haemorrhage and only chronic small vessel ischaemic change. Carotid Doppler ultrasound showed no significant stenosis and normal vertebral flow. Chest radiography was unremarkable. The patient was admitted to the stroke ward and commenced on clopidogrel (due to previous aspirin-associated gastrointestinal bleeding) and atorvastatin. He passed the initial swallow screen.

A speech and language therapy assessment identified moderately dysarthric but functional speech, with fatigability, hyponasality and difficulty with /s/ sounds, and a mild, effortful swallow with preserved airway protection. A soft or easy-to-chew diet with normal fluids, upright posture, slow rate, and sips of water between mouthfuls was recommended. There were no overt aspiration events. ENT review, including flexible nasal endoscopy, showed normal supraglottic and glottic structures with symmetrical vocal cord movement and no lesions, supporting a non-structural cause for his symptoms.

The neurology review documented a Glasgow Coma Scale score of 15/15, persistent left ptosis without ophthalmoplegia or diplopia, fluctuating and fatigable dysarthria, and normal limb strength and reflexes. In the context of normal neuroimaging, this pattern raised strong suspicion of MG. Further investigations were arranged, including serum AChR and anti-MuSK antibodies, nerve conduction studies and electromyography with repetitive nerve stimulation and planned single-fibre EMG, computed tomography of the chest to exclude thymoma, forced vital capacity (FVC) assessment, and continued speech and language therapy input.

Pyridostigmine 30 mg three times daily was commenced and increased to four times daily. Over the next 24-48 hours, there was rapid and marked improvement in ptosis, speech clarity, and swallowing. By the following day, both eyes were open, and the patient was observed reading; transient recurrence of ptosis during assessment was noted, consistent with fatigability. CT of the chest showed no thymoma. FVC measured 1.83 L, without clinical respiratory compromise.

For neurophysiological testing, pyridostigmine was briefly withheld. During this period, his unilateral ptosis and dysphagia recurred. Nerve conduction studies and limited single-fibre EMG showed no definite abnormality, with the report noting that prior treatment and technical factors might have reduced sensitivity.

Subsequent serology demonstrated positive AChR antibodies at 13.8 (above the laboratory reference range) with negative anti-MuSK antibodies. In combination with the clinical picture and reproducible response to pyridostigmine, this confirmed the diagnosis of AChR antibody-positive MG.

Oral prednisolone was introduced with stepwise escalation to 40 mg once daily, together with pyridostigmine 60 mg four times daily, lansoprazole, and bone protection. Diabetes control was monitored and treatment adjusted accordingly. Repeat speech and language therapy review showed tolerance of a normal diet and normal fluids without signs of aspiration, with the patient independently using safe swallow strategies. Ptosis had resolved and dysarthria had almost completely normalised. He was discharged once FVC remained stable and neurology review confirmed ongoing improvement, with clear advice to return urgently if swallowing, speech, or breathing worsened.

Follow-up by telephone and in clinic over the following month confirmed sustained resolution of ptosis, dysarthria, and dysphagia on pyridostigmine and prednisolone; absence of ocular, bulbar, limb, or respiratory symptoms; continued monitoring for steroid-related adverse effects and glycaemic control; and a gradual tapering plan for pyridostigmine and prednisolone. No thymoma was identified; incidental coronary calcification and mild basal lung changes were referred for routine outpatient evaluation. The final diagnosis was AChR antibody-positive MG initially presenting as a stroke mimic with unilateral ptosis and bulbar symptoms.

## Discussion

This case illustrates how MG in an older adult can closely mimic acute stroke and how unilateral ocular involvement can delay recognition of the underlying diagnosis. MG is an autoimmune disorder of the neuromuscular junction, most often associated with antibodies to the AChR, and is characterised by fluctuating muscle weakness [1]. Although MG more commonly presents before 50-60 years of age, very late-onset disease (≥65 years) is increasingly recognised, accounting for 10-15% of all cases and often associated with delayed recognition due to atypical presentation and coexisting comorbidities [1,2]. Ocular involvement is common at presentation and typically includes ptosis and diplopia; ptosis may be asymmetric or unilateral, which can lead clinicians to consider alternative focal pathologies such as third nerve palsy, Horner syndrome, or structural lesions rather than MG [1,3,4]. These diagnostic challenges are compounded by the rarity of very late-onset MG and the unusual presentation of unilateral ptosis, highlighting the importance of awareness among clinicians.

In this patient, unilateral ptosis, dysarthria, dysphagia, and vascular risk factors appropriately triggered a stroke workup. In real-world practice, when such cases present acutely, the initial priority is to rule out stroke or consider thrombolysis. Only once cerebrovascular causes are excluded can alternative diagnoses be explored through a detailed history and focused examination [5-7]. Although the subacute progression and diurnal variation were not typical for stroke, the presence of these focal neurological signs appropriately triggered urgent stroke assessment. Several features, however, were more consistent with MG than with ischemic stroke: a subacute evolution over 10-14 days rather than sudden onset, clear fatiguability and diurnal variation, normal brain and vascular imaging, and preserved limb strength and coordination. Differential diagnoses such as brainstem lesions, mitochondrial myopathies, and malignancy-related neuromuscular disorders were considered, but the clinical course and treatment response strongly supported MG as the primary diagnosis. Once stroke was excluded, MG was promptly identified based on the fatiguable, diurnal pattern of symptoms, preserved limb strength, and rapid response to pyridostigmine [1,2].

MG is a recognised stroke mimic, particularly when bulbar symptoms predominate, and similar cases have been reported where patients initially followed a stroke pathway before the neuromuscular diagnosis was made [5-7]. This case reinforces the importance of revisiting the diagnosis when clinical findings and investigations do not align with an assumed vascular event.

The clinical course also highlights the diagnostic value of symptom fluctuation and treatment response. Brief withdrawal of pyridostigmine reproduced unilateral ptosis and dysphagia, while reintroduction led to rapid improvement. Nerve conduction studies and limited single-fibre electromyography were non-diagnostic, which is well recognised in mild, treated, or technically challenging cases. Current guidance emphasises that negative or inconclusive electrophysiology does not exclude MG and supports integrating clinical features, antibody status, and therapeutic response when forming the diagnosis [4,8,9]. In this context, the positive AChR antibody result, together with a reproducible clinical pattern, provided strong confirmation of AChR antibody-positive MG despite equivocal neurophysiology.

Management in this case followed recommended practice: symptomatic treatment with pyridostigmine, early introduction of corticosteroids, exclusion of thymoma, respiratory monitoring, and structured follow-up [8-11]. Particular care was taken with steroid-related risks in an elderly man with diabetes, including gastric and bone protection and close glycaemic monitoring. His sustained remission of ocular and bulbar symptoms on this regimen demonstrates that even very late-onset disease can be effectively controlled when identified promptly.

Age-related immune changes may contribute to atypical presentations in older adults. In this patient, anti-MuSK antibody testing was performed despite positive AChR antibodies to rule out the rare possibility of double seropositive disease. This comprehensive serological evaluation ensures accurate classification, as MuSK-positive or double seropositive patients may have distinct clinical features and therapeutic implications [2-4].

Overall, this case underscores three key points. First, unilateral ptosis does not exclude MG and may be its presenting ocular sign. Second, in elderly patients with subacute, fluctuating bulbar symptoms, normal neuroimaging, and preserved limb strength, MG should be considered early as an important stroke mimic. Early recognition of fatigability, diurnal variation, and preserved limb strength, alongside timely serological or electrophysiological testing, can help avoid misdiagnosis and support prompt, appropriate treatment [8,9,12]. Third, clinicians should avoid over-reliance on a single negative electrophysiological study and instead use a combined assessment of clinical features, serology, and treatment response to reach a secure diagnosis and guide management [12].

## Conclusions

Unilateral ptosis with progressive, fluctuating dysarthria and dysphagia in an elderly patient should raise suspicion for MG, even when a stroke pathway has been initiated. Normal neuroimaging, preserved limb function, and clear symptom fatiguability are key clues that should prompt reconsideration of the diagnosis. Early consideration of MG in the differential diagnosis, supported by antibody testing and a monitored trial of pyridostigmine, followed by guideline-based immunotherapy where appropriate, can secure the diagnosis and lead to excellent outcomes, even in very late-onset disease and when neurophysiology is inconclusive. Recognition of red flags such as fatiguable, diurnal symptoms, preserved limb strength, and fluctuating ocular or bulbar deficits can facilitate timely diagnosis and appropriate management, avoiding misclassification as stroke.
